# Single strand gap repair: The presynaptic phase plays a pivotal role in modulating lesion tolerance pathways

**DOI:** 10.1371/journal.pgen.1010238

**Published:** 2022-06-02

**Authors:** Luisa Laureti, Lara Lee, Gaëlle Philippin, Michel Kahi, Vincent Pagès

**Affiliations:** Team DNA Damage and Genome Instability, Cancer Research Center of Marseille (CRCM); CNRS, Aix Marseille Univ, INSERM, Institut Paoli-Calmettes, Marseille, France; Institut Cochin, FRANCE

## Abstract

During replication, the presence of unrepaired lesions results in the formation of single stranded DNA (ssDNA) gaps that need to be repaired to preserve genome integrity and cell survival. All organisms have evolved two major lesion tolerance pathways to continue replication: Translesion Synthesis (TLS), potentially mutagenic, and Homology Directed Gap Repair (HDGR), that relies on homologous recombination. In *Escherichia coli*, the RecF pathway repairs such ssDNA gaps by processing them to produce a recombinogenic RecA nucleofilament during the presynaptic phase. In this study, we show that the presynaptic phase is crucial for modulating lesion tolerance pathways since the competition between TLS and HDGR occurs at this stage. Impairing either the extension of the ssDNA gap (mediated by the nuclease RecJ and the helicase RecQ) or the loading of RecA (mediated by RecFOR) leads to a decrease in HDGR and a concomitant increase in TLS. Hence, we conclude that defects in the presynaptic phase delay the formation of the D-loop and increase the time window allowed for TLS. In contrast, we show that a defect in the postsynaptic phase that impairs HDGR does not lead to an increase in TLS. Unexpectedly, we also reveal a strong genetic interaction between *recF* and *recJ* genes, that results in a *recA* deficient-like phenotype in which HDGR is almost completely abolished.

## Author summary

The presence of unrepaired lesions can transiently slow down or stall DNA replication which can result in the formation of a single-stranded DNA (ssDNA) gap downstream of the lesion. In order to complete replication and preserve genome stability, the ssDNA gap can be filled in by two lesion tolerance pathways, identified both in prokaryotes and eukaryotes: i) Translesion synthesis (TLS), potentially mutagenic and; ii) the error-free Homology Directed Gap Repair (HDGR) mechanism. The balance between these two pathways is important since it defines the level of mutagenesis during lesion bypass. In the present article, we show that the processing of the ssDNA gap that occurs during the presynaptic phase of the HDGR process, modulates this balance: inhibition of the ssDNA gap extension or of the recombinase loading leads to a decrease in HDGR with a concomitant increase in TLS. On the other hand, inhibition of the post-synaptic phase did not lead to any increase in TLS. We suggest that defects in the presynaptic phase affecting HDGR increase the time window allowed for TLS.

## Introduction

The genome of all living organisms is constantly damaged and some lesions can impair replication, potentially leading to mutagenesis and genome instability. Rupp and Howard-Flanders were the first to hypothesize the formation of single-stranded DNA (ssDNA) gaps on both strands in the presence of unrepaired replication-blocking lesions [1]. Since then, the discontinuous model that proposes the formation of a ssDNA gap, due to the initiation of the next Okazaki fragment (on the lagging strand) or to a repriming event (on the leading strand) has been confirmed *in vitro* and *in vivo* by several studies [2–7]. In order to complete replication and preserve cell survival, this ssDNA gap is filled in by one of the two lesion tolerance pathways, identified both in prokaryotes and eukaryotes: 1) Translesion Synthesis (TLS), which employs specialized DNA polymerases able to replicate the damaged DNA, with the potential to introduce mutations [8–10]; 2) Damage Avoidance (DA) pathways, which use the information of the sister chromatid to bypass the lesion in a non-mutagenic way through homologous recombination mechanisms [11]. In *Escherichia coli*, we have shown that in non-stressed conditions and in the absence of nucleotide excision repair, Homology Directed Gap Repair (HDGR), a DA mechanism that relies on the recombinase RecA, is the major lesion tolerance pathway employed by cells at replication-blocking lesions, while TLS pathway is a minor one [12,13]. We have also identified another lesion tolerance strategy, named Damaged Chromatid Loss (DCL) that promotes cell proliferation at the expense of losing the damaged chromatid. In particular when HDGR mechanism is impaired, cells can survive by replicating only the undamaged chromatid, allowing one of the daughter cells to survive (a pathway that could also refer to as "survival on a single chromatid") [12].

In *E*. *coli* there are two well-known RecA dependent recombinational pathways: the RecBCD pathway, involved in the repair of double strand breaks, and the RecF pathway (also known as the RecFOR pathway), involved in the repair of ssDNA gaps and required for an efficient SOS induction (reviewed in [14–16]. Originally, the RecF pathway has received less attention because most of the studies on recombination focused on processes initiated by a double strand break, such as conjugation or transduction. However, in the last decades, it became clear that this pathway, other than to be a “back-up” recombinational pathway in the absence of RecBCD, plays an important role during replication of a damaged DNA. Recently, it was shown that during non-stressed conditions, homologous recombination RecA and RecF dependent is mainly required to repair ssDNA gaps during replication other than double strand breaks [17].

The RecF pathway is evolutionary conserved in all bacteria [18] and functional orthologs are also found in Eukaria. This pathway encompasses several proteins and is divided into three distinct phases (reviewed in [15,19]: i) the presynaptic phase in which the damaged ssDNA gap is processed by different proteins (*i*.*e*. SSB, RecF, RecO, RecR, RecJ, RecQ) to promote the formation of an active RecA nucleofilament; ii) the synaptic phase in which homology pairing and strand exchange by the RecA nucleofilament occur to produce a D-loop and iii) the postsynaptic phase involving the processing of the recombinational intermediates by the resolvase RuvABC or the helicase RecG. Some of these proteins have already been well characterized *in vitro* [20–24], but their role *in vivo* and their impact on lesion tolerance pathways is not yet fully elucidated. The presynaptic phase is of particular interest since the ssDNA gap is the common substrate for both HDGR and TLS, hence how the ssDNA gap is processed to produce a RecA nucleofilament might influence the choice of lesion tolerance pathways.

Several genetic analyses indicated that the *recF*, *recO* and *recR* genes belong to the same epistasis group as the double mutants behave like the single mutants, at least for the UV sensitivity and the delay in SOS induction [25–28]. However, Henrikus *et al*. [29] recently showed that upon different DNA damage treatments, RecF and RecO have distinct spatio-temporal localizations in the cell, which could also imply different cellular functions. In particular, RecF seems to colocalize with the replisome [29]. According to *in vivo* and *in vitro* studies, it was proposed that two complexes, RecOR and RecFR are formed whose function at a ssDNA gap is to disassemble the filament of single strand binding (SSB) proteins in order to load the recombinase RecA and promote homologous recombination [28,30]. *In vitro*, the RecOR complex is sufficient to displace SSB proteins and load RecA on a ssDNA, promoting strand exchange in the absence of RecF [31,32]. On the other hand, RecF was shown to bind to gapped substrates, preferentially on the 5’ double strand-single strand (ds-ss) DNA junction [30,33] to favor RecA loading [32], and on the 3’ ds-ss DNA junction to limit RecA filament extension [34]. The UV sensitivity phenotype of the *recFOR* deficient strains is presumably due to a defect in RecA loading that leads to recombinational repair deficiency. Accordingly, a RecA mutant (*i*.*e*., the *recA730* allele) able to load itself on a ssDNA partially restores UV resistance of the *recFOR* deleted strains [35,36]. Similarly, we have shown at a single UV lesion that the *recA730* allele can partially compensate for the decrease in the HDGR level observed in a *recF* mutant [37].

The product of the *recJ* gene has also been associated with the RecF pathway [38]; however, compared to the *recFOR* deficient strains, no strong UV sensitivity phenotype was observed when the *recJ* gene was deleted [25]. Lovett and Cohen were the first to propose that *recJ* could widen the ssDNA gaps to help the formation of RecA nucleofilament or that it would help to stabilize the recombination intermediates in the post-synaptic phase [39,40]. Later, Courcelle and Hanawalt suggested that the products of *recJ* and *recQ* genes were involved in the repair of ssDNA gaps by processing the nascent lagging DNA strand during replication blockage [41,42]. The helicase RecQ (3’->5’) would help the exonuclease RecJ (5’->3’) to enlarge the ssDNA at the 5’ end of the gap to lengthen the substrate for RecA filament formation. *In vitro* data showing that SSB protein is able to stimulate the activity of RecQ and of RecJ corroborate this hypothesis [24]. Recently, we suggested that in the presence of an unrepaired lesion, RecJ is required to enlarge the ssDNA gap not only in the lagging strand but also in the leading strand [43], thus providing further evidence for the *in vivo* role of RecJ.

In this study, we explored the role of all the genes involved in the presynaptic phase of the RecF pathway, their genetic interaction and their effect on lesion tolerance pathways. The presynaptic phase appears to play an important role in the regulation of lesion tolerance, in particular the competition between TLS and HDGR: a defect in RecA loading (mediated by the RecFOR complexes) or in enlarging the ssDNA gap (mediated by RecQ and RecJ) results in HDGR impairment that is partially compensated by an increase in TLS. On the contrary, inactivation of the post-synaptic phase (resolution or dissolution of the Holliday junction) that also affects HDGR did not lead to any increase in TLS. Interestingly, our data also revealed a strong genetic interaction between *recF* and *recJ* (as well as *recF* and *recQ*): loss of the two genes results in an additive effect that almost completely abolishes HDGR similarly to a *recA* deficient phenotype. Altogether our results indicate that both the size of the ssDNA gap and the loading of RecA play an important role in the formation of an efficient D-loop structure, thus favoring the non-mutagenic HDGR mechanism over the error-prone TLS pathway.

## Results

**Fig 1:**
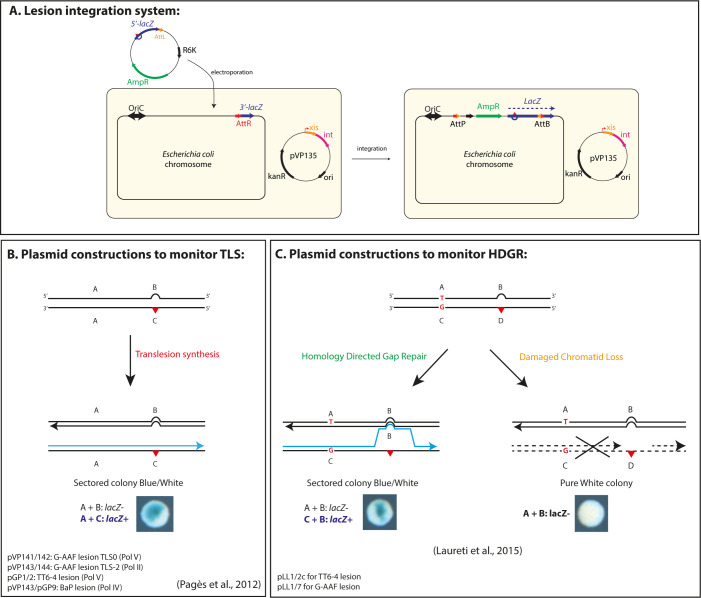
Lesion tolerance assay and lesion containing plasmids. **A)** The system is based on the phage lambda site-specific recombination and it requires a recipient *E*. *coli* strain with a single *att*R site fused to the 3’-end of the *lacZ* gene and a non-replicating plasmid construct containing a single DNA lesion (red triangle) in the 5’-end of the *lacZ* gene fused to the *att*L site. The recombination reaction between *att*L and *att*R is controlled by ectopic expression of phage lambda *int–xis* proteins (provided by pVP135), and leads to the integration of the lesion-containing vector into the chromosome. Integrants are selected on the basis of their resistance to ampicillin and the chromosomal integration restores an entire *lacZ* gene (see also [44]). **B)** Plasmid duplexes used to monitor TLS events (see also [13,52]). The non-damaged strand (A+B markers) contains a short sequence of heterology opposite the lesion that inactivates the *lacZ* gene and serves as a genetic marker for strand discrimination. Only the replication by TLS polymerases of the A+C markers will give rise to a functional *lacZ* gene and therefore to a sectored blue-white colony. For the G-AAF lesion, one construct measures TLS0 events specific of Pol V-dependent TLS events, while another one contains a +2 frameshit that allows to measure TLS-2 events specific of Pol II when the G-AAF lesion is inserted in the *NarI* hotspot sequence (see also [51]). **C)** Plasmid duplexes used to specifically monitor strand exchange mechanisms, *i*.*e*. HDGR events (see also [12]). The system is a modified version of the plasmid constructs used to monitor TLS events. In these plasmids four genetic markers have been designed in order to distinguish the replication of the non-damaged strand (containing markers A and B) from the damaged strand (containing markers C and D). Using a combination of frameshift and stop codon, we inactivated *lacZ* gene on both the damaged (C-D) and undamaged (A-B) strands of the vector. Only a strand exchange mechanism by which replication has been initiated on the damaged strand (incorporation of marker C), and where a template switch occurred at the lesion site (leading to incorporation of marker B) will restore a functional *lacZ* gene (the combination of markers C and B contains neither a stop codon nor a frameshift); therefore leading to sectored blue-white colonies. When the damaged strand is lost, only the A and B marker will be replicating giving rise to a white colony.

In the present work, we used a previously described assay that allows to insert a single replication-blocking lesion into the chromosome of *E*. *coli* and to monitor lesion tolerance mechanisms by a colorimetric assay based on the *lacZ* reporter gene [12,13,44]. Briefly, a non-replicative plasmid containing a defined DNA lesion in the 5’-end of the *lacZ* gene is inserted into a precise locus of *E*. *coli* genome reconstituting the entire *lacZ* gene (Fig 1A). Plasmid integration is irreversible and is achieved by the site-specific recombination system of the bacteriophage lambda, integration events are selected using an antibiotic resistance marker. Using different types of lesion-containing plasmids, we can insert the lesion either on the lagging or in the leading orientation regarding the replication fork direction and directly monitor either TLS (Fig 1B) or HDGR mechanisms (Fig 1C). In the data presented below, we did not observe any difference in the level of HDGR or TLS between leading and lagging strand, in line with previous data from Fijalkowska laboratory that proposed that a DNA lesion has the same probability to block replication and to be bypassed whether it is on the lagging or the leading strand [45]. *E*. *coli* possesses three TLS polymerases, involved in lesion bypass: Pol II (encoded by *polB*) [46], Pol IV (encoded by *dinB*) [47] and Pol V (encoded by *umuDC*) [48]. Under physiological conditions, Pol II and Pol IV are already present in the cell, however, the expression level of all three TLS polymerases increases upon SOS induction [49,50]. UV lesions (*i*.*e*. the cyclobutane pyrimidine dimer, CPD, and the thymine-thymine pyrimidine (6–4) pyrimidone photoproduct, TT6-4) are bypassed by Pol V, but in non-stressed conditions this event is very rare (<0.5%) [13]. The chemical guanine adduct N-2-acetylaminofluorene (G-AAF) when positioned in the *Nar*I hotspot sequence, can be bypassed by Pol V in an error-free manner (TLS0 events), or by Pol II, inducing a -2 frameshift (TLS-2 events) [8,51]. The chemical guanine adduct benzo(a)pyrene (BaP) is bypassed by Pol IV [8]. We have constructed specific lesion integration plasmids to monitor their bypass by each of these TLS polymerases (See Fig 1B).

The *E*. *coli* strains used in this study are deficient for the mismatch repair system (*mutS*) to prevent correction of the genetic markers of the integrated plasmids, as well as for nucleotide excision repair (*uvrA*), to avoid excision of the lesion and to focus on lesion tolerance events (see also [12,13]).

### Loading of the recombinase RecA: role of the mediators

**Fig 2:**
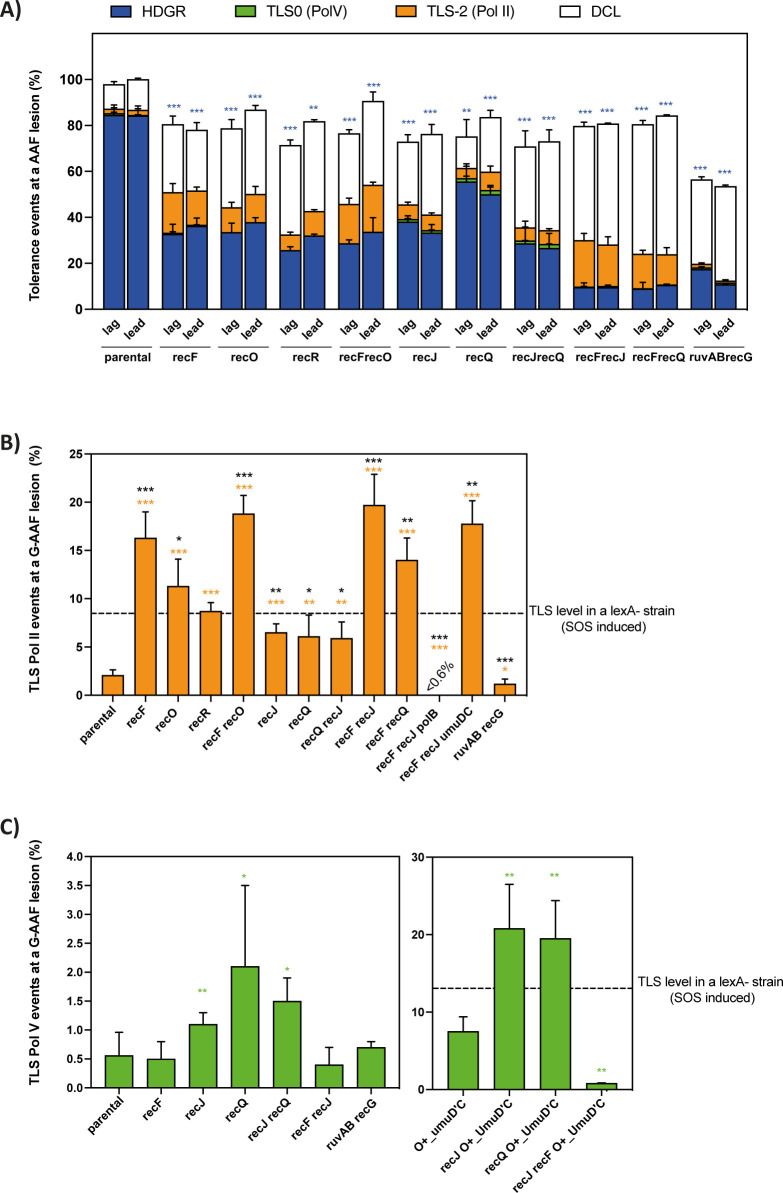
Lesion tolerance events in the presence of a G-AAF lesion. **A)** The graph represents the partition of lesion tolerance pathways (*i*.*e*. HDGR, TLS and DCL) and the cell survival in the presence of a G-AAF lesion for the strains affected in the presynaptic and postsynaptic phase of the RecF pathway (for more details see Material and Methods section). The lesion was inserted either in the lagging (lag) or in the leading (lead) strand compared to the replication fork direction, however no statistically significant differences were found between leading and lagging strands. The blue asterisks represent the statistical significance for HDGR events for every mutant strain compared to the parental strain. **B)** The graph represents the percentage of Pol II TLS events in the presence of a G-AAF lesion. The data for lagging and leading have been pooled together. In the graph we indicate the level of Pol II TLS for *lexA* strain, as previously measured in [70]. The orange and the black asterisks represent the statistical significance for Pol II TLS events for every mutant strain compared to the parental strain or the *lexA* strain, respectively. **C)** The graph represents the percentage of Pol V TLS events in the presence of a G-AAF lesion. The data for lagging and leading have been pooled together. In the graph we indicate the level of Pol V TLS for *lexA* strain, as previously measured in [70]. The green asterisks represent the statistical significance for Pol V TLS events for every mutant strain compared to the parental strain. O+_UmuD’C is a strain expressing an already cleaved form of UmuD under its own promoter. The data in every graph represent the average and standard deviation of at least three independent experiments. Statistical analyses have been performed with Prism using an unpaired *t*-test. * p < 0,05; ** p < 0,005; *** p < 0,0005. #: The data for *recF* and *recJ* strains have been already published in [12,43].

Previously, we have shown that deletion of *recF* gene strongly reduced HDGR, while increasing the Pol II TLS events for the G-AAF lesion and Pol IV TLS events for the BaP lesion [12,52]. In the absence of its mediators (*i*.*e*. RecF, RecO or RecR), RecA loading can still occur, but randomly and with a slower kinetics [53,54]. Since HDGR relies on the recombinase RecA [12], this mechanism is necessarily affected. Similarly, induction of the SOS response is affected by a delay in RecA nucleofilament formation [26,27,55]. The delay in RecA filament formation also explains why we measured a very low level of Pol V TLS events (<0.5%) in a *recF* strain [12,52] (See also Fig 2C). Indeed, to be active Pol V requires interaction with the tip of a RecA nucleofilament assembled by RecFOR complex [56–58].

RecF together with RecO and RecR are considered as the recombination mediator proteins of RecA and the three genes are thought to be epistatic, even if in the last decades some studies suggested that their cellular functions could be distinct [29,32]. To further investigate the role of the recombination mediator genes and their genetic interaction during lesion bypass, we inactivated each of them and monitor lesion bypass using the G-AAF lesion that allows to monitor both Pol II and Pol V mediated TLS. Deletion of either *recF* [12], *recO* or *recR* (Fig 2A) resulted in a strong decrease in HDGR, accompanied by a strong increase in Pol II dependent TLS events (Fig 2B). The level of Pol II TLS in the *recF* strain is two-fold higher than the one observed in a *lexA* deficient strain where the SOS system is fully induced (dotted line in Fig 2B). The double mutant *recF recO* showed no further decrease in the level of HDGR compared to the single mutants *recF*, *recO* or *recR*, neither a significant increase in the level of Pol II dependent TLS compared to *recF* (Fig 2A and 2B). We have observed a similar decrease in HDGR for *recO* and *recF recO* strains for the UV TT6-4 lesion [37]. Altogether our genetic data indicate that at least for lesion bypass, the *recF*, *recO* and *recR* genes belong to the same epistasis group and that the three genes play an important role in HDGR, which is expected due to their role in mediating RecA loading.

### Effect of the ssDNA gap processing on lesion bypass

We have recently suggested that the size of the ssDNA gap modulates lesion tolerance pathways. Lack of the nuclease RecJ could result in a shorter RecA-covered ssDNA gap, thus homology search and strand exchange are less efficient, impacting HDGR mechanism on one hand and favoring TLS pathway on the other hand. This was shown for Pol II TLS events at the G-AAF lesion [43]. Similarly, we observed an increase in Pol IV TLS at the BaP lesion in the absence of *recJ* (S1A Fig).

The helicase RecQ has been proposed to help RecJ in enlarging the ssDNA gap at the 5’ end [41]. We deleted the *recQ* gene and monitored lesion tolerance pathways in the presence of both G-AAF and UV TT6-4 lesions. We observed a decrease in HDGR, but lower compared to *recJ* or *recF* deletion (Figs 2A and S1B). This might indicate that even if the helicase activity of RecQ is required to provide the substrate for the nuclease activity of RecJ, it is not essential or that another helicase, yet to be identified, can partially compensate for the loss of RecQ, as also proposed by Courcelle *et al*. [42]. Deletion of both *recJ* and *recQ* in the presence of either a G-AAF or a TT6-4 lesion showed the same level of HDGR and TLS as the single mutant *recJ* (Figs 2A and S1B). Hence, from our genetic analyses, *recJ* and *recQ* appeared to belong to the same epistasis group for lesion bypass, however RecJ plays a more critical role in HDGR mechanism.

More interestingly, for these strains we measured a more than 2-fold increase in Pol V TLS events (Fig 2C). To corroborate these data, we inactivated *recJ* or *recQ* in a strain that expresses an allele of pre-activated Pol V where the UmuD subunit is already cleaved in UmuD’ (indicated as O+_UmuD’C). We again observed a strong increase in Pol V dependent TLS events (Fig 2C) which indicates that impairing the extension of the ssDNA gap favours replication by TLS polymerases. In these strains, unlike in the *recF-O-R* strains, RecA loading and nucleofilament formation can still occur efficiently allowing the contact with Pol V that is required for its activation [56,57]. As expected, in the absence of RecF, there is no increase in Pol V dependent TLS as shown by the mutant strains *recF recJ* and *recF recJ O+_UmuD’C* (Fig 2C). For the TT6-4 lesion, the decrease in HDGR in the *recJ*, *recQ* and *recJ recQ* strains was not compensated by any increase in Pol V TLS (S1B Fig). This is most likely due to the low level of Pol V and because Pol V requires elevated dNTP levels to bypass a TT6-4 lesion [59]. Indeed, even in a *lexA* deficient strain where SOS is fully induced, the level of TLS at this lesion is only ~2% [52]. In line with our hypothesis, Courcelle and coworkers observed in UV irradiated *E*. *coli* cells, in which SOS is induced and the level of dNTPs is strongly increased, that Pol V was required for survival in the absence of *recJ* or *recQ* and that the increase of mutagenesis was Pol V dependent [42,60].

### Additive effect of *recF* and *recJ* genes

From our data, it appears that both RecA loading and the size of the ssDNA gap are important factors to modulate the partition and the efficiency of lesion tolerance pathways. To further investigate the presynaptic phase, we decided to analyze the genetic interaction between *recF* and *recJ* (and between *recF* and *recQ*), by deleting both genes and monitoring lesion bypass in the presence of a G-AAF and TT6-4 lesion. Interestingly, for both lesions the double mutants showed a drastic drop in HDGR events (resulting in less than 10% of HDGR), compensated mainly by damaged chromatid loss (or survival on a single chromatid) (Figs 2A and S1B), similar to what we have previously observed in a *recA* deficient strain [12]. The 10% of sectored blue-white colonies still observed when measuring HDGR in the *recF recJ* (and *recF recQ)* strain as well as in the *recA* deficient strain result from a still uncharacterized RecA-independent mechanism, as previously proposed by the Lovett and Rosenberg laboratories [17,61].

These data revealed that regarding the HDGR mechanism, *recF* and *recJ* (or *recQ*) genes are not epistatic as it was expected since they are part of the same recombinational RecF pathway [38]. Regarding TLS, such additive effect is not observed for Pol II TLS events at a G-AAF lesion since the level is similar to that observed in the *recF* and *recF recO* strains (Fig 2B). Likewise, no significant increase in Pol IV dependent TLS was observed when we deleted both *recF* and *recJ* genes in the presence of a BaP lesion (S1A Fig). We confirmed that the TLS events measured were exclusively Pol II dependent, by inactivating *polB* or *umuDC* genes in the double mutant *recF recJ* (Fig 2B). Compared to a *recA* strain, the double mutants *recF recJ* (and *recF recQ)* can still perform TLS because even in the absence of RecF, RecA loading can still occur (but less efficiently) and this is sufficient to help stabilizing the ssDNA gap favoring replication by Pol II TLS (or Pol IV). Similarly, a better stability of the ssDNA gap could explain why we did not observe a drastic decrease in cell survival for the double mutant *recF recJ* as in a *recA* deficient strain. Altogether these results indicate that both the loading of RecA and the size of the ssDNA gap are equally important for an efficient HDGR or else TLS will take over.

### Competition between TLS and HDGR does not occur at the postsynaptic phase

Our results reveal that the presynaptic phase plays an important role in modulating lesion tolerance pathways: when HDGR is reduced, the level of TLS increases. We wondered whether we could extend this concept to the post-synaptic phase of the RecF pathway. After its formation, the RecA nucleofilament will engage in homology search and strand exchange, producing a D-loop structure typical of the HDGR mechanism. Once the ssDNA gap is filled in most likely by the DNA polymerase Pol I or Pol III [62,63], the postsynaptic phase employs either the resolvase RuvABC or the helicase RecG to process the recombinational intermediates [64–67]. Deletion of either *ruv* genes or *recG* gene have only a modest impact on recombination proficiency, while the double mutant *ruvABC recG* is extremely sensitive to DNA damage and recombination is severely affected [68], indicating a functional redundancy between RecG and RuvABC. Therefore, we inactivated both *ruvAB* and *recG* genes and monitored lesion tolerance pathways in the presence of a G-AAF lesion. As expected, HDGR was strongly affected as well as cell survival (Fig 2A), similar to a *recA* strain [12]; however, no concomitant increase in TLS was observed neither by Pol II nor by Pol V (Fig 2B and 2C). This indicates that the competition between TLS and HDGR does not occur at the postsynaptic phase: once the RecA filament engages in strand exchange, forming the D-loop structure, TLS pathways cannot take place anymore.

### The TLS increase in the presynaptic mutants is not correlated to SOS induction

In a previous study, we have already shown that affecting HDGR favors TLS: mutant alleles of RecA, that can form a nucleofilament but are impaired for homology search and strand exchange, strongly favor lesion bypass by TLS polymerases [52]. However, in this particular context, SOS response was constitutively activated thus the amount of TLS polymerases in the cells was significantly increased, which contributed to shifting the balance towards TLS pathway [52]. We therefore wondered whether the increase in TLS observed when the presynaptic genes are inactivated was also dependent on the activation of the SOS response. In *E*. *coli* the SOS response is negatively regulated by the transcriptional repressor LexA: upon DNA damage ssDNA accumulates and is rapidly covered by RecA. Such RecA nucleofilaments will then stimulate LexA self-cleavage, thus inducing the SOS system and activating roughly 40 different genes, among them the genes coding for RecA and the TLS polymerases [69].

**Fig 3:**
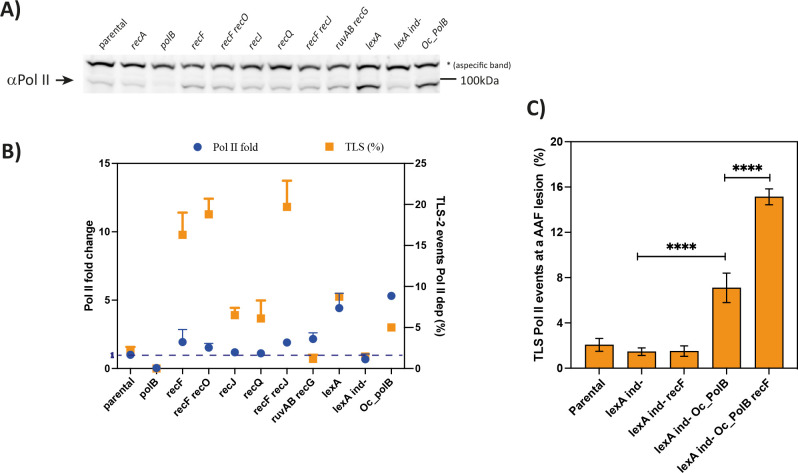
The presynaptic phase modulates TLS pathway independently from SOS induction. **A)** Western blot measuring the expression level of Pol II. As negative control *polB* deficient strain was used, while the *lexA* strain was used as positive control. The asterisk indicates an aspecific band recognized by the anti-Pol II antibodies. **B)** Graph correlating the Pol II fold change with the percentage of Pol II TLS events measured with our *in vivo* genetic system. The fold changes, compared to our parental strain, were quantified by Image Lab software (Biorad) and represent the average and the standard deviation of 2–3 independent experiments (see also S2A Fig). NB: For some dots the error bars are not visible because they are too small. **C)** The graph represents the percentage of Pol II TLS events in the presence of a G-AAF lesion. The data for lagging and leading have been pooled together and represent the average and standard deviation of at least three independent experiments. Statistical analyses have been performed with Prism using an unpaired *t*-test. *** p < 0,0005. *LexA ind-* indicates a strain in which a specific mutation prevents LexA cleavage and therefore the SOS regulon can never be induced. *Oc_polB* indicates a strain in which *polB* is expressed under a constitutive promoter.

To evaluate the level of SOS induction, we measured by immunoblotting the level of Pol II in the mutants of the presynaptic phase. For some strains, we observed an increase in Pol II, however, the level of Pol II remains very low compared to the one observed in a *lexA* deficient strain in which SOS is fully and constitutively activated (Figs 3A and S2A). In the same strains, we also measured an increase in RecA level (S2B Fig), confirming an induction of the SOS response. In parallel, we measured with our genetic system the Pol II TLS events using a specific allele of *lexA* that cannot be cleaved (named *lexA ind-*), thus preventing SOS induction. To our surprise, in a *lexA(ind-) recF* deficient strain, in the presence of the G-AAF lesion the Pol II TLS level drops drastically to a level similar to the *lexA(ind-)* strain (Figs 3C and 2B). This result seems to further suggest that the induction of the SOS response is necessary for the TLS increase when the presynaptic genes are affected. However, we could not find any correlation between the measured level of Pol II TLS and the expression levels of Pol II (and RecA—Figs 3B and S2C). For instance, a high level of Pol II (and RecA), as in a *lexA* strain, results in a rather low level of TLS-2 (~8%) compared to a moderate level of Pol II expression in the *recF*, *recF recO* and *recF recJ* strains where the level of TLS is high (15 to 20%) (see also Fig 2B). More convincingly, *ruvAB recG* strain showed an increase in Pol II and RecA levels similar to a *recF* and *recJ* strain, but no increase in Pol II TLS events was measured (Figs 3B and S2C).

From these results, we hypothesized that a minimal cellular level of Pol II was required to observe an increase in TLS when the presynaptic phase is affected. Indeed, in the *lexA(ind-)* strain the level of Pol II decreases of roughly 40% (Figs 3A and S2A) and this could explain why no increase in Pol II TLS events is measured even if when *recF* is inactivated (Fig 3C). In order to test this hypothesis, we used a strain in which *polB* is under a constitutive promoter (named Oc_PolB), therefore its expression is high and independent from SOS regulation [70]. As expected, constitutive expression of Pol II in a *lexA ind-* strain results in an increase in TLS events, but more interestingly inactivation of the presynaptic gene *recF* in the *lexA ind- Oc-PolB* strain (where the level of Pol II is constant and not SOS-regulated) results in a further increase, similar to what observed in a *recF* strain (Figs 3C and 2B). This result indicates that affecting the presynaptic phase by affecting the efficiency of HDGR leads to an increase in TLS, independently of the mild SOS induction that we have measured. The only requirement is a substantial level of Pol II similar to the one measured in our parental strain.

## Discussion

The use of our single lesion assay allows to finely explore what occurs in the presence of a single replication blocking lesion and to better decipher the regulation of lesion tolerance pathways. This work evinces how the presynaptic phase of the recombinational RecF pathway plays a key role in lesion tolerance pathway choice, defining the time window allowed for TLS to compete with HDGR. Once the presynaptic phase achieved, the RecA nucleofilament will engage in homology search and strand pairing, resulting in a D-loop structure that promotes non-mutagenic pathway (HDGR), while preventing error prone pathway (TLS) in the so called post-synaptic phase. Our data show that defects in the genes of the presynaptic phase affect HDGR mechanism, leading to a significant increase in TLS pathways that has no correlation with the mild SOS induction observed in the mutant strains. In the absence of either RecJ or RecQ, the ssDNA gap is most likely not enlarged, thus impairing the efficiency of HDGR mechanism. Likewise, deletion of the RecA mediators (*recF*, *recO* or *recR*) causes a significant delay in RecA nucleofilament formation, as shown *in vitro* where nucleation time goes from 2–10 min in the presence of RecFOR to 10–60 min in its absence [53]. In both cases, HDGR is severely affected and we observed an increase in TLS pathways.

**Fig 4:**
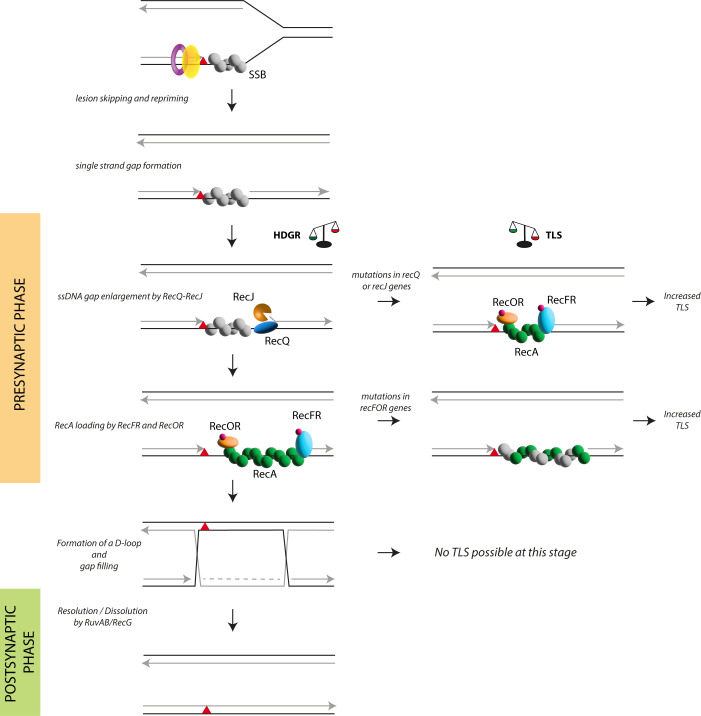
The presynaptic phase of the RecF pathway modulates lesion tolerance pathways. Here is represented a scheme of single strand gap repair, with a particular focus on the presynaptic phase of the RecF pathway. A replication-blocking lesion (red triangle) is positioned on the leading strand. During replication, unrepaired lesions can temporarily block the replicative polymerase. The polymerase can skip the lesion and restart replication downstream, leaving a ssDNA gap that is immediately covered by SSB proteins. From this moment on, the presynaptic phase of the RecF pathway takes place: first the helicase RecQ and the nuclease RecJ are loaded at the 5’ ds-ssDNA junction to enlarge the size of the gap, then the RecFR complex together with the RecOR complex are recruited to displace SSB and load the recombinase RecA in the 5’->3’ direction on the ssDNA gap. The presynaptic phase ends with the formation of a RecA nucleofilament that will engage in homology search and strand exchange forming a D-loop structure, which in turn leads to the final step of the HDGR mechanism, the post synaptic phase. The competition between HDGR and TLS can occur only during the presynaptic phase: mutations affecting *recQ* or *recJ* genes will impact the efficiency of HDGR because of the smaller size of ssDNA gap, in favor of the TLS polymerases. Similarly, in the absence of the RecF, RecO or RecR, RecA loading will occur randomly and the kinetics of filament formation will be slower, resulting in a decrease in HDGR mechanism and in an increase in Pol II and Pol IV TLS. The combination of RecF and RecJ (or RecQ) deficiency has an additive effect on HDGR similar to the one observed in the *recA* deficient strain, since the ssDNA gap is smaller and the loading of RecA strongly delayed. Impairing the postsynaptic phase also reduces HDGR but does not lead to an increase in TLS.

Based on these data, we propose a model that illustrates how the choice of lesion tolerance pathways is directed during the presynaptic phase of the RecF pathway (Fig 4). In the presence of an unrepaired lesion, the replicative polymerase temporarily stalls before a repriming event allows replication to continue, leaving a ssDNA gap. SSB protein will immediately cover the ssDNA gap to protect it and to orchestrate the recruitment of several proteins. SSB was indeed shown to directly interact with most of the proteins of the presynaptic phase as well as with the TLS polymerases [71–73] and the SSB-coated ssDNA gap can be regarded as a platform for protein recruitment for the processing of the ssDNA gap and for lesion bypass. The 5’ ds-ssDNA junction of the ssDNA gap appears to be a shared substrate for both RecQJ and RecFR complexes. Whether and which factors regulate the timing of RecQJ and RecFR recruitment is not known yet, however, we suggest that RecQ and RecJ are the first to be recruited at the 5’ ds-ssDNA junction of the nascent DNA. We based this hypothesis on the fact that RecQ and RecJ were shown to directly interact with SSB in the presence of a ssDNA gap and this interaction stimulates their catalytic activity to enlarge the ssDNA gap [24,74–76]. Supporting our hypothesis, Xia and coworkers [17] showed that RecJ and RecQ promote recombination and Holliday junction formation in vegetative *E*. *coli* cells, acting upstream of RecA during the repair of ssDNA gaps. Therefore, the extension of the gap must come before the loading of RecA. We have previously estimated the size of the ssDNA gaps to be in the range of 1.8–3.5 Kb [43] which correlates with the processivity established *in vitro* for RecJ [75]. Once the ssDNA gap has been enlarged by the RecQJ complex, the RecFR complex will be firstly recruited to the 5’ ds-ssDNA junction (as also suggested by [32]) followed by the recruitment of the RecOR complex to the ssDNA gap where RecO directly interacts with SSB [77,78]. Indeed, while *in vitro* RecOR complex is sufficient to load RecA on a ssDNA, RecF is needed in the presence of a gapped ssDNA [30,32], hence our *in vivo* data showing the requirement of RecF suggests the presence of ssDNA gaps at the lesion site. The presence of RecFR to the ssDNA gap will prevent RecQJ complex to be recruited again and further extend the gap, to a point where HDGR mechanism might be affected. Indeed, in the *recF* strain it was observed a RecJ-dependent degradation of the nascent DNA that caused a delay in replication resumption [79,80]. Finally, RecA loading and filament formation can start thanks to the concerted action of RecFR-RecOR complexes. Once the RecA nucleofilament on the ssDNA gap is formed it will engage in homology search and strand invasion, forming the so-called D-loop structure. From this moment on, the time window for TLS is closed and lesion bypass can only occur through the HDGR mechanism. For this reason, inhibition of HDGR during the post-synaptic phase, as in the *ruvAB recG* deficient strain, does not have any effect on TLS mechanism.

Other than the nature of the DNA lesion, we have already shown that several factors contribute to regulate the balance between HDGR and TLS. In bacteria, TLS can be favored by inducing the SOS response that increases the amount of the TLS polymerases in the cell [70]. We have previously shown that the proximity of lesions on opposite strands can generate overlapping ssDNA regions due to the action of RecJ leading to an increase in TLS [43]. Finally, in this work, we show that the presynaptic phase also plays a role in the regulation of lesion tolerance pathways.

These findings, if confirmed in eukaryotic cells, could open the way to new strategies in cancer treatment: targeting specifically the post-synaptic phase of homologous recombination could enable to sensitize the cells to chemotherapies without allowing TLS to contribute to the survival of the cells, nor increasing mutagenesis.

## Material and methods

### Bacterial strains and growth conditions

All *E*. *coli* strains used in this work are listed in S1 Table. They are either derivative of strains FBG151 and FBG152 [13], that carry a plasmid that allows the expression of the *int–xis* genes after IPTG induction, or derivative of strains EC1 and EC2 (this study), in which the *int–xis* genes have been inserted into the chromosome of *E*. *coli*. Strains were grown on solid and in liquid Lysogeny Broth (LB) medium. Gene disruptions were achieved by the one-step PCR method [81] or by P1 transduction using the Keio collection [82]. Following the site-specific recombination reaction, the lesion is located either in the lagging strand (FBG151 or EC1 derived strains) or in the leading strand (FBG152 or EC2 derived strains). Antibiotics were used at the following concentrations: ampicillin 50 or 100 μg/ml; tetracycline 10 μg/ml, kanamycin 100 μg/ml, chloramphenicol 30 μg/ml. When necessary, IPTG and X-Gal were added to the medium at 0.2mM and 80 μg/ml, respectively.

### Plasmids

pVP135 expresses the integrase and excisionase (*int–xis*) genes from phage lambda under the control of a *trc* promoter that has been weakened by mutations in the -35 and the -10 region. Transcription from Ptrc is regulated by the *lac* repressor, supplied by a copy of *lacI*^*q*^ on the plasmid. The vector has been modified as previously described [13].

pVP146 is derived from pACYC184 plasmid where the chloramphenicol resistance gene has been deleted. This vector, which carries only the tetracycline resistance gene, serves as an internal control for transformation efficiency.

pVP141-144 and pGP1, 2 and 9 are derived from pLDR9-attL-lacZ as described in [13]. pLL1, pLL2c and pLL7 are derived from pVP141 and contain several genetic markers as previously described [12]. All these plasmid vectors contain the following characteristics: the ampicillin resistance gene, the R6K replication origin that allows plasmid replication only if the recipient strain carries the *pir* gene, and the 5’ end of the *lacZ* gene in fusion with the *attL* site-specific recombination site of phage lambda. The P’3 site of *att*L has been mutated (AATCATTAT to AATTATTAT) to avoid the excision of the plasmid once integrated. These plasmids are produced in strain EC100D pir-116 (from Epicentre Biotechnologies, cat# EC6P0950H) in which the pir-116 allele supports higher copy number of R6K origin plasmids. Vectors carrying a single lesion for integration were constructed as previously described following the gap-duplex method [13]. A 13-mer oligonucleotide, 5′-GCAAGTTAACACG-3′, containing no lesion or a TT(6–4) lesion (underlined) in the *Hinc*II site was inserted either into the gapped-duplex pLL1/2c leading to an out of frame *lacZ* gene (to measure HDGR) or into the gapped-duplex pGP1/2 leading to an in frame *lacZ* gene (to measure TLS0). A 15-mer oligonucleotide 5’-ATCACCGGCGCCACA-3’ containing or not a single G-AAF adduct (underlined) in the *Nar*I site was inserted into the gapped-duplex pLL1/7 (to measure HDGR) or into the gapped-duplexes pVP141-142 or pVP143-144 to score respectively for TLS0 Pol V-dependent and for TLS-2 Pol II-dependent. A 13-mer oligonucleotide, 5′-GAAGACCTGCAGG, containing no lesion or a dG-BaP(-) lesion (underlined) was inserted into the gapped-duplex pVP143/pGP9 leading to an in frame *lacZ* gene (to measure TLS).

All new batch of plasmid constructions are validated by integration in the parental strain to verify the concentration adjustment.

### Monitoring HDGR and TLS events

The protocol for lesion integration assay is described in details in [37,44]. Cells were diluted and plated before the first cell division using the automatic serial diluter and plater EasySpiral Dilute (Interscience) and were counted using the Scan 1200 automatic colony counter (Interscience). No differences were observed when we used FBG or EC derivative strains.

For every integration experiment, we used the lesion versus non-lesion plasmid constructions, each plasmid solution containing an equal amount of pVP146 plasmid as internal control. Following the integration of the pLL1/2c vector (TT6-4 lesion) or pLL1/7 vector (AAF lesion), sectored blue/white colonies represent HDGR events. Following integration of the pVP141/142, pVP143/144, pGP1/2, pVP143/pGP9 vectors (G-AAF, TT6-4 or BaP lesion, respectively), sectored blue/white colonies represent TLS events. The relative integration efficiencies of lesion-carrying vectors compared with their lesion-free homologues, normalized by the transformation efficiency of pVP146 plasmid in the same electroporation experiment, allow the overall rate of lesion tolerance to be measured (which corresponds to the cell survival in the presence of a single lesion). In the parental strain one lesion gives about 100% cell survival. For each lesion we combine the results obtained with the different plasmids to measure HDGR and TLS events (for BaP lesion we only measure TLS events). Briefly, the mean of the absolute values (blue colonies/total colonies) of HDGR and TLS events were multiplied by the mean of the cell survival obtained with all the plasmids construction for a given lesion. Indeed, whether TLS or HDGR was measured, cell survival will not change for a given lesion. The value of DCL was calculated by subtracting the HDGR and TLS values to the total cell survival.

Tolerance events (Y axis) represent the percentage of cells able to survive in presence of the integrated lesion compared to the lesion-free control. The data in every graph represent the average and standard deviation of at least three independent experiments of a lesion inserted in the leading (or in the lagging) orientation. Statistical analyses were done using GraphPad Prism applying an unpaired *t*-test.

### Protein extraction and western blot analysis

The strains of interest were grown and processed as for the integration of the plasmid with a lesion. After electroporation, cells were left to recover at 37°C during 45 min, then harvested, and resuspended in 100–200μl of lysis buffer (50 mM Tris HCl pH7.5, 100 mM KCl, 1 mM EDTA, 1 mM DTT, 10% glycerol, 0,1% NP40, 1mM PMSF, 200 ug/mL lysozyme freshly made). After 30 min incubation on ice, cells were sonicated 10 min using the Bioruptur (set up at high, 0.5 ON/0.5 OFF), spin down for 30 min at 4°C and the supernatants stored at −80°C. Total protein quantification was determined using the BCA Kit (Thermo).

Samples were diluted in ratio 1:1 with Laemmli sample buffer (62.5 mM Tris–HCl pH 6.8, 25% glycerol; 2% SDS; 5% β-mercaptoethanol; bromophenol blue), boiled at 95°C and resolved in precast Bolt 4–12% Bis-Tris plus gel (Thermo) with MOPS buffer. Semi dry transfer in precast Transblot Turbo membrane 0.2 um nitrocellulose (Biorad) was done.

For RecA immunoblot 200ng of total protein extract was loaded and primary antibodies from Abcam (ab63797) were used at a 1:3000 dilution. For Pol II immunoblot 20 μg of total protein extract was loaded and primary antibodies kindly provided by Mark Sutton were used at a 1:10000 dilution. Secondary antibodies anti-rabbit 700 or 800 (Biorad) were used at a 1:10000. A Chemidoc MP imaging system (Biorad) was used for signal detection and Image Lab software (Biorad) was used for quantification.

## Supporting information

S1 Table FBG151 = MG1655 ΔlacIZ ΔattBλ::attRλ-3’lacZ-aadA. (lesion inserted in the lagging orientation compared to replication). FBG152 = MG1655 ΔlacIZ ΔattBλ::3’lacZ-attRλ-aadA. (lesion inserted in the leading orientation compared to replication). EC1 = MG1655 ΔlacI-lacZ::frt ΔattBλ::attRλ-3’lacZ-aad intergenic(aqpZ-ybjD)::high pTRC λ int xis lacIq (lesion inserted in the lagging orientation compared to replication). EC2 = MG1655 ΔlacI-lacZ::frt ΔattBλ::3’lacZ-attRλ-aad intergenic(aqpZ-ybjD)::high pTRC λ int-xis lacIq (lesion inserted in the leading orientation compared to replication).
(PDF)

S1 Fig A) The graph represents the percentage of Pol IV dependent TLS events in the presence of a BaP lesion. The data for lagging and leading have been pooled together. The violet asterisks represent the statistical significance for Pol IV TLS events for every mutant strain compared to the parental strain. B) The graph represents the partition of lesion tolerance pathways (i.e. HDGR, TLS and DCL) and cell survival in the presence of the UV TT6-4 lesion. The lesion was inserted either in the lagging (lag) or in the leading (lead) strand compared to the replication fork direction, however no statistically significant differences were found between leading and lagging strands. The blue asterisks represent the statistical significance for HDGR events for every mutant strain compared to the parental strain. The data in every graph represent the average and standard deviation of at least three independent experiments. Statistical analyses have been performed with Prism using an unpaired t-test: *** p < 0,0005.
(PDF)

S2 Fig A) Quantification of Pol II fold change expression level (compared to the parental strain). The data represent the average and standard deviation of 2–3 independent experiments. B) Western blot measuring the expression level of RecA. As negative control recA deficient strain was used, while the lexA strain was used as positive control. Below the quantification of RecA fold change compared to the parental strain. The data represent the average and standard deviation of 2–3 independent experiments. C) Graph correlating the RecA fold change with the percentage of Pol II TLS events measured with our in vivo genetic system.
(PDF)
