# Supplementary material for: Single strand gap repair: The presynaptic phase plays a pivotal role in modulating lesion tolerance pathways
Source: PLoS Genet. 2022 Jun 2;18(6):e1010238. doi: 10.1371/journal.pgen.1010238 (PMC9203016; doi:10.1371/journal.pgen.1010238)
Supplement: S1 Table — (lesion inserted in the lagging orientation compared to replication). FBG152 = MG1655 ΔlacIZ ΔattBλ::3’lacZ-attRλ-aadA. (lesion inserted in the leading orientation compared to replication). EC1 = MG1655 ΔlacI-lacZ::frt ΔattBλ::attRλ-3’lacZ-aad intergenic(aqpZ-ybjD)::high pTRC λ int xis lacIq (lesion inserted in the lagging orientation compared to replication). EC2 = MG1655 ΔlacI-lacZ::frt ΔattBλ::3’lacZ-attRλ-aad intergenic(aqpZ-ybjD)::high pTRC λ int-xis lacIq (lesion inserted in the leading orientation compared to replication). (PDF) [file pgen.1010238.s001.pdf]

S1 Table : *E. coli* strains used in this study

Name	Short name	Genotype
EVP22/23	Parental strain	FBG151/152 *uvrA*::frt *mutS*::frt
EVP146/147	*recO*	FBG151/152 *uvrA*::frt *mutS*::frt *recO::*frt
EVP195/196	*recQ*	FBG151/152 *uvrA*::frt *mutS*::frt *recQ* ::frt
EVP210/211	*Oc_polB*	FBG151/152 *uvrA::frt mutS::frt frt::Oc_polB*
EVP223/224	*O+_UmuD'C*	FBG151/152 *uvrA*::frt *mutS*::frt frt::*o+_umuD'C*
EVP602/603	*recO recF*	FBG151/152 *uvrA*::frt *mutS*::frt *recO::*frt *recF::*Kan
EVP347/348	*recJ*	FBG151/152 *uvrA*::frt *mutS*::frt *recJ::frt*
EVP357/358	*O+_UmuD'C recJ*	FBG151/152 *uvrA*::frt *mutS*::frt *recJ::frt* frt::*o+_umuD'C*
EVP359/360	*O+_UmuD'C recQ*	FBG151/152 *uvrA*::frt *mutS*::frt *recQ::frt* frt::*o+_umuD'C*
EVP433/434	*ruvAB recG*	FBG151/152 *mutS::frt uvrA::frt ruvAB::frt recG::frt*
EVP498/499	*phrB recF*	FBG151/152 *uvrA*::frt *mutS*::frt *phrB* ::frt *recF* ::frt
EVP588/589	*recJ recF*	FBG151/152 *uvrA*::frt *mutS*::frt *recJ*::frt *recF* ::frt
EVP615/616	*recQ recF*	FBG151/152 *uvrA*::frt *mutS*::frt *recQ* ::frt *recF* ::frt
EVP712/713	*lexA ind-*	FBG151/152 *uvrA::frt mutS::frt LexA (ind-)*
EVP963/964	*recQ recJ*	FBG151/152 *uvrA*::frt *mutS*::frt *recQ* ::frt *recJ::frt*
EVP975/976	*recF recJ polB*	FBG151/152 *uvrA::frt mutS::frt polB::frt recF::Cm recJ::frt*
EVP981/982	*recF recJ O+_UmuD'C*	FBG151/152 *uvrA*::frt *mutS*::frt *recJ::frt* frt::*o+_umuD'C* *recF::frt*
EVP993/994	*recR*	FBG151/152 *uvrA*::frt *mutS*::frt *recR*::frt
EVP989/990	*phrB recF recO*	FBG151/152 *uvrA*::frt *mutS*::frt *phrB* ::frt *recF*::frt *recO* ::frt
EVP1006/1007	*lexA ind- recF*	FBG151/152 *uvrA::frt mutS::frt LexA (ind-) recF::frt*
EVP1010/1011	*recF recJ umuDC*	FBG151/152 *uvrA::frt mutS::frt recJ::frt recF::frt umuDC::frt*
EVP1016/1017	*lexA ind- Oc_polB*	FBG151/152 *uvrA::frt mutS::frt LexA (ind-) Oc_PolB::frt*
EVP1018/1019	*lexA ind- recF Oc_polB*	FBG151/152 *uvrA::frt mutS::frt LexA (ind-) recF::frt Oc_PolB::frt*
EC13/14	Parental strain	EC1/EC2 *uvrA*::frt *mutS*::frt
EC39/40	*recO*	EC1/EC2 *uvrA*::frt *recO::*frt *mutS*::frt
EC49/50	*recO recF*	EC1/EC2 *uvrA*::frt *recO::*frt *mutS*::frt *recF*::frt
EC51/52	*recF*	EC1/EC2 *uvrA*::frt *recF::*frt *mutS*::frt

FBG151 = MG1655 ΔlacIZ ΔattBλ::attRλ-3'lacZ-aadA (lesion inserted in the lagging orientation compared to replication)
FBG152 = MG1655 ΔlacIZ ΔattBλ::3'lacZ-attRλ-aadA (lesion inserted in the leading orientation compared to replication)
EC1 = MG1655 ΔlacI-lacZ::frt ΔattBλ::attRλ-3'lacZ-aad intergenic(aqpZ-ybjD)::high pTRC λ int-xis lacIq
EC2 = MG1655 ΔlacI-lacZ::frt ΔattBλ::3'lacZ-attRλ-aad intergenic(aqpZ-ybjD)::high pTRC λ int-xis lacIq
