# Supplementary material for: Single strand gap repair: The presynaptic phase plays a pivotal role in modulating lesion tolerance pathways
Source: PLoS Genet. 2022 Jun 2;18(6):e1010238. doi: 10.1371/journal.pgen.1010238 (PMC9203016; doi:10.1371/journal.pgen.1010238)
Supplement: S1 Fig — A) The graph represents the percentage of Pol IV dependent TLS events in the presence of a BaP lesion. The data for lagging and leading have been pooled together. The violet asterisks represent the statistical significance for Pol IV TLS events for every mutant strain compared to the parental strain. B) The graph represents the partition of lesion tolerance pathways (i.e. HDGR, TLS and DCL) and cell survival in the presence of the UV TT6-4 lesion. The lesion was inserted either in the lagging (lag) or in the leading (lead) strand compared to the replication fork direction, however no statistically significant differences were found between leading and lagging strands. The blue asterisks represent the statistical significance for HDGR events for every mutant strain compared to the parental strain. The data in every graph represent the average and standard deviation of at least three independent experiments. Statistical analyses have been performed with Prism using an unpaired t-test: *** p < 0,0005. (PDF) [file pgen.1010238.s002.pdf]

S1 Figure

A)

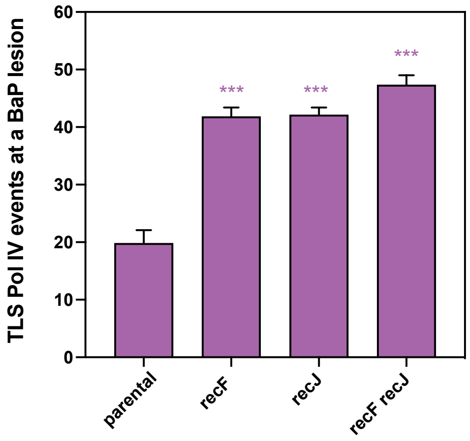

B)

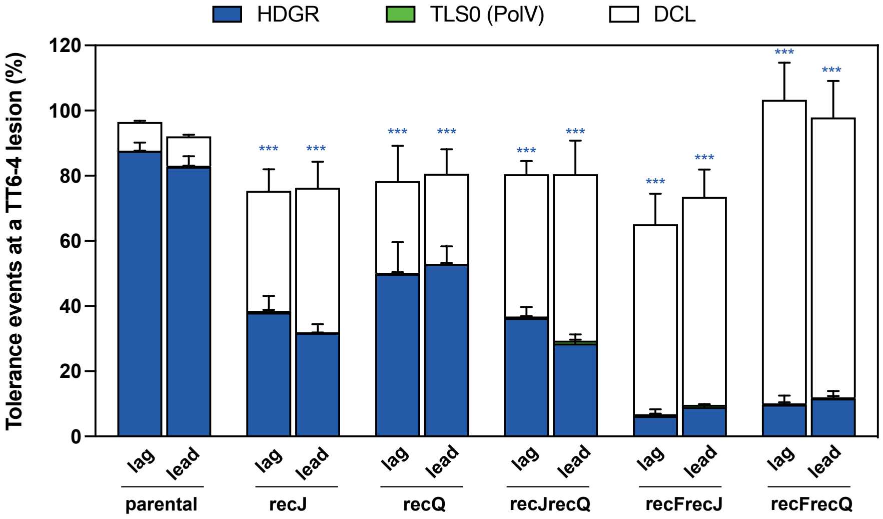

S1 Figure.

A) The graph represents the percentage of Pol IV dependent TLS events in the presence of a BaP lesion. The data for lagging and leading have been pooled together. The violet asterisks represent the statistical significance for Pol IV TLS events for every mutant strain compared to the parental strain

B) The graph represents the partition of lesion tolerance pathways (i.e. HDGR, TLS and DCL) and cell survival in the presence of the UV TT6-4 lesion. The lesion was inserted either in the lagging (lag) or in the leading (lead) strand compared to the replication fork direction, however no statistically significant differences were found between leading and lagging strands. The blue asterisks represent the statistical significance for HDGR events for every mutant strain compared to the parental strain.

The data in every graph represent the average and standard deviation of at least three independent experiments. Statistical analyses have been performed with Prism using an unpaired t-test: *** p < 0,0005
