# Supplementary material for: Single strand gap repair: The presynaptic phase plays a pivotal role in modulating lesion tolerance pathways
Source: PLoS Genet. 2022 Jun 2;18(6):e1010238. doi: 10.1371/journal.pgen.1010238 (PMC9203016; doi:10.1371/journal.pgen.1010238)
Supplement: S2 Fig — A) Quantification of Pol II fold change expression level (compared to the parental strain). The data represent the average and standard deviation of 2–3 independent experiments. B) Western blot measuring the expression level of RecA. As negative control recA deficient strain was used, while the lexA strain was used as positive control. Below the quantification of RecA fold change compared to the parental strain. The data represent the average and standard deviation of 2–3 independent experiments. C) Graph correlating the RecA fold change with the percentage of Pol II TLS events measured with our in vivo genetic system. (PDF) [file pgen.1010238.s003.pdf]

S2 Figure

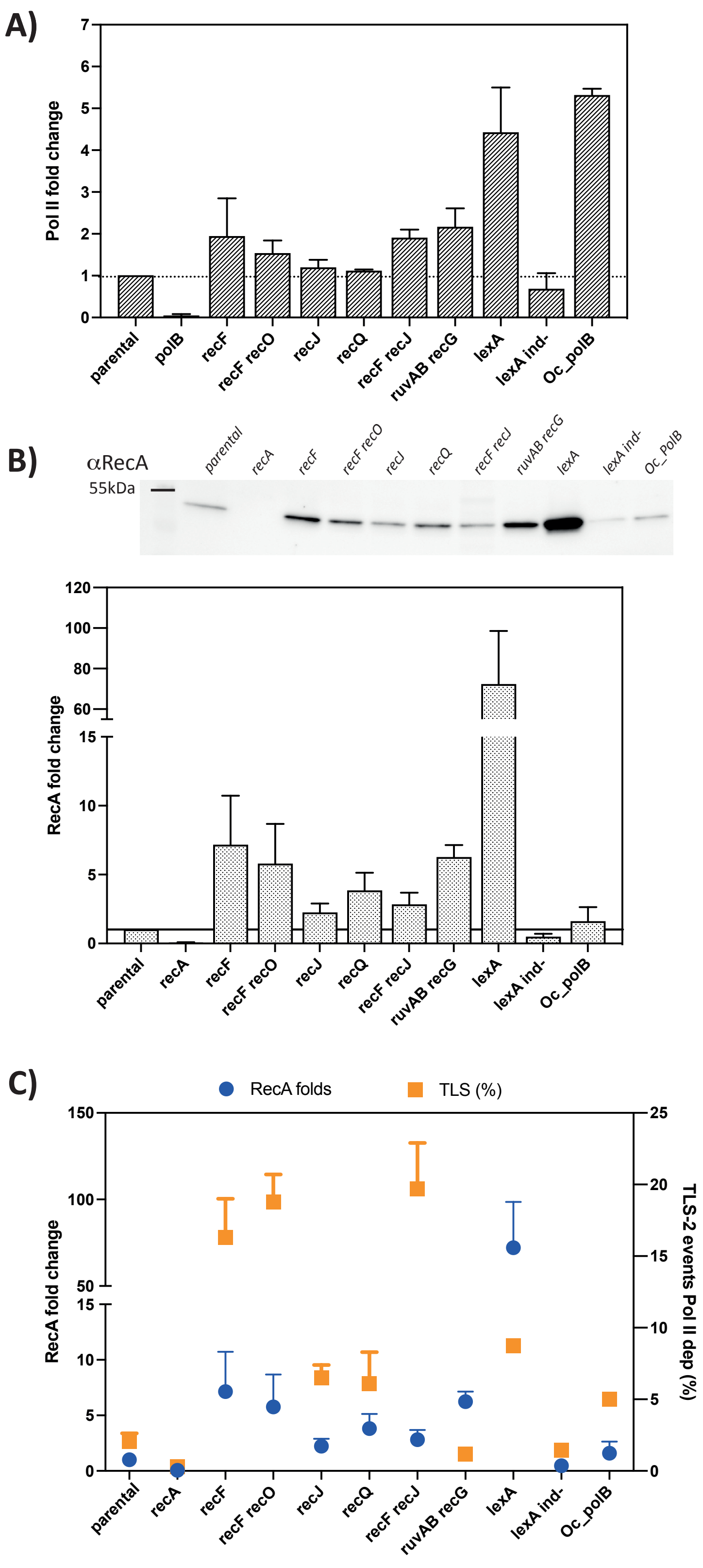

S2 Figure :
A) Quantification of Pol II fold change expression level (compared to the parental strain). The data represent the average and standard deviation of 2-3 independent experiments.
B) Western blot measuring the expression level of RecA. As negative control recA deficient strain was used, while the lexA strain was used as positive control. Below the quantification of RecA fold change compared to the parental strain. The data represent the average and standard deviation of 2-3 independent experiments.
C) Graph correlating the RecA fold change with the percentage of Pol II TLS events measured with our in vivo genetic system.
